# L Test Subtask Segmentation for Lower-Limb Amputees Using a Random Forest Algorithm

**DOI:** 10.3390/s24154953

**Published:** 2024-07-31

**Authors:** Alexis L. McCreath Frangakis, Edward D. Lemaire, Helena Burger, Natalie Baddour

**Affiliations:** 1 Department of Mechanical Engineering, Faculty of Engineering, University of Ottawa, Ottawa, ON K1N 6N5, Canada; nbaddour@uottawa.ca; 2 Faculty of Medicine, University of Ottawa, Ottawa, ON K1H 8M5, Canada; elemaire@ohri.ca; 3 University Rehabilitation Institute, University of Ljubljana, 1000 Ljubljana, Slovenia; helena.burger@ir-rs.si; 4 Faculty of Medicine, University of Ljubljana, 1000 Ljubljana, Slovenia

**Keywords:** L test, subtask segmentation, wearable sensor, random forest, machine learning, Timed Up and Go

## Abstract

Functional mobility tests, such as the L test of functional mobility, are recommended to provide clinicians with information regarding the mobility progress of lower-limb amputees. Smartphone inertial sensors have been used to perform subtask segmentation on functional mobility tests, providing further clinically useful measures such as fall risk. However, L test subtask segmentation rule-based algorithms developed for able-bodied individuals have not produced sufficiently acceptable results when tested with lower-limb amputee data. In this paper, a random forest machine learning model was trained to segment subtasks of the L test for application to lower-limb amputees. The model was trained with 105 trials completed by able-bodied participants and 25 trials completed by lower-limb amputee participants and tested using a leave-one-out method with lower-limb amputees. This algorithm successfully classified subtasks within a one-foot strike for most lower-limb amputee participants. The algorithm produced acceptable results to enhance clinician understanding of a person’s mobility status (>85% accuracy, >75% sensitivity, >95% specificity).

## 1. Introduction

**Figure 1:**
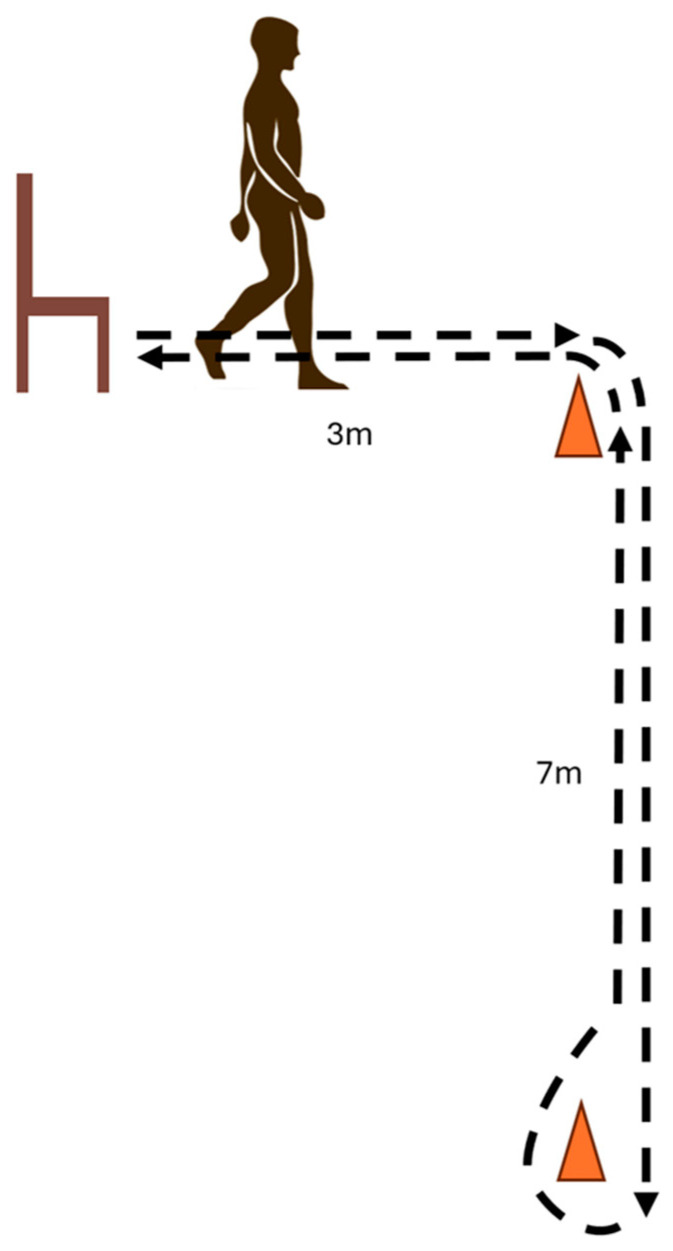
Diagram of the L test.

The L test of functional mobility can provide information regarding a person’s ability to safely ambulate and participate in tasks of everyday life, especially for lower-limb amputees (LLAs) [1]. The L test was designed to overcome the limited sensitivity and ceiling effects of the commonly used functional mobility test, the Timed Up and Go [1]. Within the L test, patients must complete a circuit where they stand up from a chair, walk to a marker, turn 90°, walk towards a second marker, turn 180°, walk back to the first marker, turn 90°, walk back to the chair, and sit-down (Figure 1). Each individual movement (stand-up, sit-down, turn, or walk) is considered a subtask.

While the L test traditionally only provides the total time to complete the circuit, clinicians would benefit from additional information on the various subtasks [2]. Previous research has shown a significant difference in standing up after lower limb amputation [3,4,5,6]. In 2018, Caronni et al. observed that turning parameters could be a good predictor of balance measures [7] and, in 2021, Williams et al. showed that patients with dementia displayed slower subtask times and turning velocity than when compared to the results of able-bodied individuals [8]. Additionally, the Amputee Mobility Predictor, which is a test used to predict activity level in amputees, includes stand-up, sit-down, and 180° turns, with observational balance a major scoring point on the assessment [9]. Therefore, it would be useful to identify how these subtasks might benefit lower-limb amputee patients and how clinicians may use them to guide treatment decisions.

**Figure 2:**
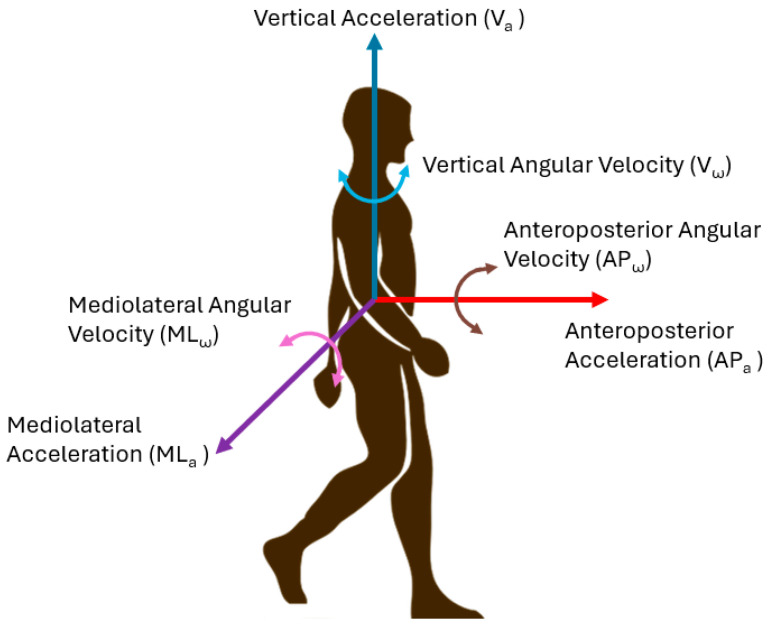
Parametric directions are used in inertial data.

Inertial measurement unit (IMU) instrumented subtask segmentation of similar functional mobility tests has been analyzed in the literature [2,10], with at least 15 studies assessing wearable sensor-based subtask segmentation of the Timed Up and Go (TUG) [11,12,13,14,15,16,17,18,19,20,21,22,23,24,25] and nine studies using subtask segmentation to inform clinical diagnosis or recovery planning [26,27,28,29,30,31,32,33,34]. IMU sensors in smartphone devices are ubiquitous, with five of the 24 studies using these integrated sensors [10]. Smartphone sensor data include linear acceleration, angular velocity, rotation angle (Figure 2), and azimuth (i.e., angle about the vertical axis from true north). Smartphones are a convenient device to use for L test data collection because they are easily accessible (i.e., globally, most clinicians will own or have access to a smartphone). Smartphones can automatically perform all calculations, provide outcome measures immediately after the trial [35], and possibly use wireless communication to securely transfer outcome measures to a data repository.

Smartphone positioning on the individual affects movement detection. The most common location for a single IMU is the lower back [21,23,24,25,26,27,29,30,32] because this location gives a good approximation of the user’s center of mass. The center of mass acceleration from IMU data has good to excellent reliability for tests such as the Sensory Organization Test and could identify individuals with varying balance conditions [36].

While IMU segmentation has been completed for other functional mobility tests, the L test still requires research on segmentation [10]. Previous research demonstrated good results for rule-based L test segmentation in able-bodied individuals [37]; however, the algorithm used a generalized approach [38] that did not translate well when tested on a lower-limb amputee population [39], which is a recommended population for this test [1]. The rule-based algorithm did not obtain high sensitivity, likely due to problems classifying the altered, and often slower, mobility pattern that occurs in many lower-limb amputees [39]. Therefore, further investigation on L test subtask segmentation is required for the intended population. More complex algorithms using machine learning approaches may be required to produce suitable results [40]. One other published algorithm partially segmented walk and turn subtasks within the L test [41]; however, this preliminary research had only five participants who were all unilateral transtibial amputees. Further investigation with a larger dataset is needed to train and assess models segmenting all subtasks for the L test when completed by lower-limb amputees [1].

One study showed good results for fall risk classification from the 6-Minute Walk Test (6MWT) with IMU data and a random forest algorithm [1,2]. This random forest fall risk model worked well with 129 lower-limb amputee participants with a single smartphone IMU. Additionally, random forest algorithms have been used to classify wearable IMU data for other applications [42,43,44]. These results suggest that a random forest algorithm may be a suitable approach for TUG or L test segmentation for addressing the data from amputee participants.

This paper reports on a random forest algorithm for segmenting the L test for people with a lower limb amputation. An algorithm for lower-limb amputee L test analysis could provide a basis for further assessing clinical measures such as fall risk and rehabilitation progress [10]. Information regarding individual subtasks may allow clinicians to identify imbalances during walking or turning and provide an objective outlook on individual ability [10].

## 2. Materials and Methods

### 2.1. Participants

Data were collected from a convenience sample of six male and 15 female able-bodied participants between the ages of 19 and 68 (average age: 36 ± 19 years) and 15 male and 10 female lower-limb amputee participants between the ages of 29 and 86 (average age: 58 ± 14 years) with an average time since amputation of 11 ± 17 years (range of one to 61 years). Exclusion criteria included individuals with cognitive issues that affected their ability to follow instructions. Participants provided informed consent prior to participating. Able-bodied participant data collection was approved by the University of Ottawa’s Office of Research Ethics and Integrity (H-09-22-8351). Lower-limb amputee data collection was approved by the University Rehabilitation Institute of the Republic of Slovenia Soča Ethics Council (035-1/2021-3.4). Participant characteristics are shown in Table 1 and Table 2.

### 2.2. Data Collection

**Figure 3:**
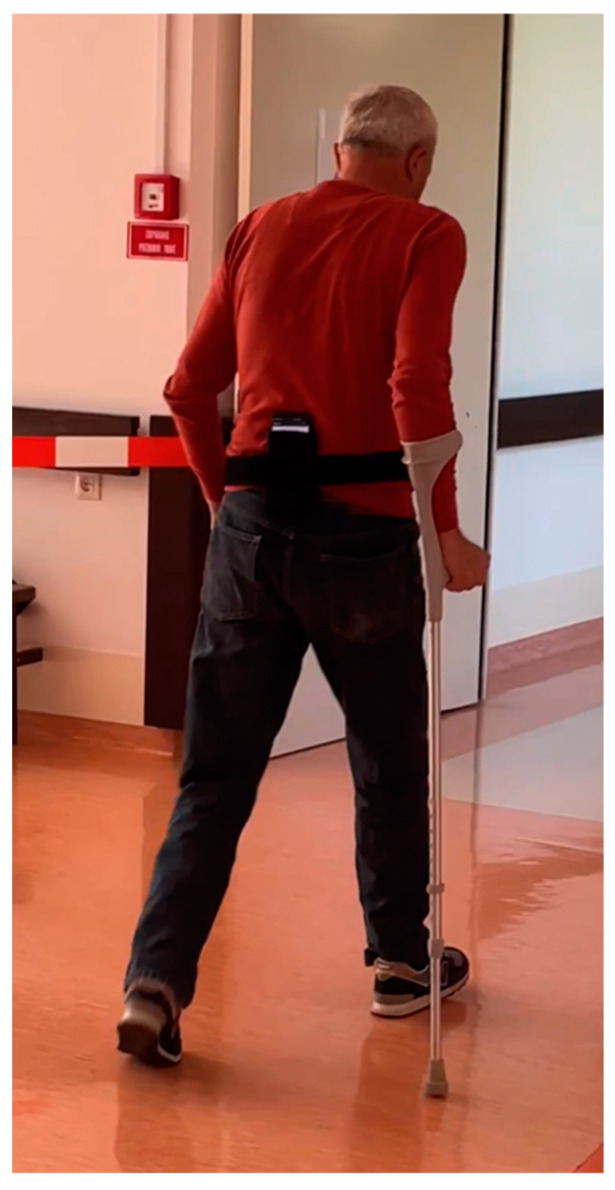
Lower-limb amputee participant completing an L test trial.

A custom belt was fastened around the waist of each participant; the belt secured a Samsung Galaxy S10+ Smartphone (Samsung Electronics Canada, Mississauga, ON, Canada) in a posterior pocket (Figure 3). This posterior-pelvis position was chosen for its approximation of the center of mass as well as its demonstrated efficacity in other algorithms [35]. A custom app recorded IMU data at 60 Hz, including raw and linear accelerations in the mediolateral, anteroposterior, and vertical directions; rotation angle in the mediolateral, anteroposterior, and vertical directions; azimuth angle; and angular velocity in the mediolateral, anteroposterior, and vertical directions. Participants were instructed to complete the L test at a walking speed that was fast but safe according to their own capabilities. Able-bodied participants completed five trials each. Lower-limb amputee participants completed one trial each. The app provided an auditory cue to indicate that the recording had begun; participants were then informed that they could begin the first trial. Once each trial was completed, the researcher would inform the participant that this was the end of the current trial and that the participant could begin the next trial.

### 2.3. Ground Truth

A second smartphone (Apple iPhone XR, iOS 17) was used to video record participants at a sample rate of 60 Hz as they completed the tests. Ground truth times of events of interest were determined from the video using the VSDC Free Video Editor v9.1 [45].

Initiation of the stand-up task was defined as the beginning of trunk flexion, and termination corresponded to maximal trunk extension, whether this occurred before or after the first step. The reversal of these actions defined the beginning and end of the sit-down task. Turn initiation was identified as the beginning of pelvis rotation, and turn completion was the end of this rotational movement.

### 2.4. Data Processing

Raw L test data from smartphone IMU sensors were imported into a custom-built Python program. Initially, the algorithm performed an angle correction on the azimuth signal [35], which corrected jumps in the signal that occur when a participant turns past 360° or 0° [35]. A threshold technique identified changes in azimuth magnitude greater than 10° between data points and, if true, added or subtracted this magnitude change from the signal [35].

Ground truth timestamps of the initial three foot strikes of each trial were determined. These were selected due to their clear acceleration peaks, allowing alignment of the ground truth times and inertial data. Acceleration and angular velocity data were filtered using a fourth-order zero-lag Butterworth low-pass filter with a 4 Hz cut-off frequency [10,17,35].

### 2.5. Datasets

Individual models were trained for each of the six subtests, specifically stand-up, sit-down, first 90° turn, second 90° turn, first 180° turn, and second 180° turn subtasks. To train the six models, the corresponding approximate data ranges were extracted for each subtask as a preprocessing step. Data ranges are shown in Table 3. For each subtask, the models were only trained and tested on the corresponding extracted set of data. This subtask data extraction decreased the large class imbalance that occurs when the complete trial data are used in model training. To ensure that important data were not removed, the earliest and latest array index for each subtask were identified across all trials and all participants (Table 3). These overall indices were applied to each trial when extracting subtask data. The datasets for each subtask included a ground truth column, which contained one-hot encoded data for the indices for which the subtask was occurring (1) or not occurring (0). After this data extraction, there were six data files for each participant trial, one for each subtask (stand-up, first 90° degree turn, first 180° turn, second 90° turn, second 180° turn, and sit-down). Each of these datasets contained 11 columns: system time; acceleration in the mediolateral, vertical, and anteroposterior axes; angular velocity about the mediolateral, vertical, and anteroposterior axes; rotation angle about the mediolateral and anteroposterior axes; azimuth; and one-hot encoded ground truth.

### 2.6. Feature Extraction

Feature sets for mean, standard deviation, variation, maximum value, minimum value, range, kurtosis, and skewness were created for each of the nine IMU signals using a window size of 24 (0.4 s) [21,22,46]. These features were also calculated for a version of the data set that was reversed, since this was shown to be effective in previous algorithms. Linear acceleration and angular velocity signals were magnitude-normalized [10,18]. The total number of features for each data file was 82. To increase algorithm efficiency, ineffective features were removed using Sci-Kit Learn’s feature selection function, f_classif. This method computes the ANOVA F value for each feature.

### 2.7. Classification Technique

SciKitLearn’s Random Forest Classifier was applied to the datasets for each subtask [47]. Each subtask had its own classifier that was trained on the subtask-specific dataset, for a total of six classifiers. The default maximum depth parameter was used, where all nodes expand until either all leaves were pure or they contain the minimum number of samples [47]. The default criterion used was Gini impurity. A variety of tree sizes (50, 100, 250, 500, 750, 1000) were assessed with each of the feature sets (5, 10, 15, 25, 50, 75, 82) using SciKitLearn’s hyperparameter optimization function, GridSearchCV [48]. The GridSearchCV function completes an exhaustive search for the highest accuracy (using sklearn.metric.accuracy_score) after a 5-fold cross-validation [48]. Hyperparameter optimization output is the number of trees and a corresponding number of features with the highest accuracy. To assess whether a chronologically forward or reverse direction of analysis was better for a particular subtask, the GridSearchCV function was used on both forward and reverse data, and the direction that produced the best result was chosen. The GridSearchCV results are tabulated in Table 4. The algorithm was trained on both lower-limb amputee data and able-bodied data. Testing was implemented using a leave-one-out (LOO) strategy with only the lower-limb amputee data because the objective of the current study is to identify if the algorithm could successfully segment movements completed by lower-limb amputees. Outcome measures included accuracy, specificity, and sensitivity.

### 2.8. Data Analysis

The criteria for successful classification were to identify the transition between subtasks within one average step time for the participant under testing. The average time step was calculated for each participant and was unique to that participant. To determine this period, the average step time for each participant was calculated from the ground truth data. For successful classification, the absolute difference between an algorithm and ground truth times must be less than the average step time for that participant. The percentage of participants in which this successful clarification occurred is referred to as Accuracy_AD_. Additionally, to assess successful classification, the overall accuracy, sensitivity, and specificity of each of the models were assessed.

## 3. Results

Table 4 shows the Accuracy_AD_ results for the beginning and end of each subtask, along with the output for the optimized number of trees and features from the GridSearchCV function and the most successful direction of analysis. Table 5 shows the ANOVA F feature correlation results used to assemble the lists of features for algorithm training. Only the top ten features for each subtask are shown for the direction of analysis mentioned in Table 4. Table 6 shows the overall accuracy, specificity, and sensitivity for each subtask.

## 4. Discussion

An algorithm was designed to automatically segment L test subtasks when completed by individuals with lower limb amputations, using data from smartphone IMU sensors. This algorithm successfully classified subtasks within a one-foot strike for most participants. The algorithm produced acceptable results to enhance the clinician’s understanding of a person’s mobility status.

As with similar subtask segmentation studies, identifying the ground truth start and end of subtask transitions was often difficult [14,23]. For example, the beginning and end of turns, especially for individuals with a slower gait, can occur over multiple steps. Therefore, there was ambiguity when defining the ground truth for the beginning and end of tasks. In many studies, including the current study, a single researcher labeled all ground truth time stamps; however, some studies used the average of multiple reviewers. For assessing AI classification, being within one participant step is likely sufficient for assessing the person’s movements during the subtasks, which gives allowance for this common ambiguity in labeling. For clinical interpretation of these results, future research is needed to evaluate clinically meaningful differences for times greater than one step.

The ANOVA F feature correlation showed that the most correlated feature for all turn subtasks was the azimuth signal’s range, followed by the azimuth signal’s standard deviation. Features derived from the azimuth and gyroscope Y signals were most common for all turning subtasks, while features derived from the linear acceleration x, gyroscope x, and pitch signals were most common for stand-up and sit-down subtasks. This is aligned with parameters used for almost all rule-based or machine learning algorithms for TUG test segmentation [10]. System time only appeared in the top ten features for the first 90° turn. This may have been due to the preprocessing of the data into separate files for each subtask; therefore, the time that the tasks were completed was less relevant than if the model was given the full array of data for the entire test and had to differentiate between, for example, the first and second 90° turns.

This is the first study to segment stand-up, sit-down, and all-turn subtasks within the L test using data from people with lower limb amputations. Previous research that used IMUs for mobility analysis reported differences between able-bodied and amputee populations [28], thereby demonstrating the importance of producing models specifically for this population. The current study showed that the random forest algorithm is viable for subtask segmentation of lower-limb amputee inertial data. Since no other published research is available for amputee full L test segmentation, the following paragraphs compare outcomes from this study to able-bodied or other population results in the literature for other functional mobility tests.

Pew et al. [41] observed the possibility of segmenting turn intent for the L test completed by an LLA population using a single IMU. The highest accuracy was from a k-nearest neighbor (kNN) model that predicted turns with 76% accuracy. Pew et al. also had 96% accuracy with a support vector machine (SVM) model; however, this used an approach described as “individual training”, where accuracies were obtained from models that were trained on the data from the person under investigation. While this approach can lead to higher accuracy, it is not realistic in a clinical setting, where patient data would not be available to train a new model for each clinical encounter. The current study was evaluated using a leave-one-out method and obtained higher accuracy than the model assessed by Pew et al.

There were several studies that used a single IMU to segment similar functional mobility tests on varying populations. De Luca et al. [22] trained a k-means clustering algorithm on 2-Minute Walk Test (2MWT) and 5 Times Sit to Stand (5STS) data, with the most successful sensor achieving overall accuracies of 84% for stand-up and 72% for turning (20 healthy adults). Results for sit-downs were not discussed. The current study surpassed these accuracy values. Hellmers et al. [21] obtained accuracies of 99% for turnaround, sit-to-stand, and stand-to-sit of the TUG using a single IMU-instrumented belt. Sensitivity for these subtasks was 78% for turnaround, 84% for stand-up, and 94% for sit-down. The current study was able to obtain higher sensitivities for 180° turns, which were the only turns assessed in the Hellmers et al. study. The current algorithm was not able to classify the sit-down task as successfully as the algorithm designed by Hellmers et al. (148 elderly) [21]. Abdollah et al. [19] used head-mounted IMUs with a rule-based approach to segment stand-up and sit-down subtasks with the TUG and obtained an accuracy of 93% for stand-up and 99% for a sit-down, a sensitivity of 90% for stand-up and 98% for a sit-down, and a specificity of 96% for stand-up and 100% for sit-down (12 healthy adults). The current algorithm obtained similar accuracy and specificity but 21% lower sensitivity.

Overall, the current study was often able to achieve better results than previous studies (with differing populations) for turns. However, our proposed approach had variable success for stand-up and sit-down subtasks when compared to other studies.

Several studies demonstrated better results at the cost of using additional IMUs. This included Nguyen et al. [14], who achieved 100% sensitivity and specificity for all subtasks using an optimal selection of signals from 17 sensors placed around the body. Hseih et al. [46] investigated a variety of machine learning techniques to classify TUG subtasks. An adaptive boosting algorithm obtained 84.84% sensitivity for the stand-up subtask, 89.07% for the second 180° turn subtask, and 80.84% for the sit-down subtask. A decision tree algorithm obtained 87.42% sensitivity for the first 180° turn. These results were obtained using six IMUs. While the use of multiple IMUs can sometimes provide more sensitive results, it is important to consider the financial, accessibility, and setup time requirements of using multiple IMUs. With our proposed approach, all a clinician would need is a smartphone and the time to put a belt on the patient, start the app, and put the app in the belt pocket.

The stand-up and sit-down tasks were the most successful, with Accuracy_AD_ scores of 96% for both the beginning and end of the stand-up task and 92% for both the beginning and end of the sit-down subtask. The beginning of the stand-up subtask showed consistent results when analyzed in the forward direction, among all depths, trees, and features. The end of the stand-up subtask gave the best results at a depth of five and above 250 trees. At higher depths, the algorithm did not perform as well for this subtask.

The first 90° turn was classified with a higher overall Accuracy_AD_ (92% for the beginning of the subtask, 76% at the end) than the second 90° turn (92% for the beginning of the subtask, 72% at the end). The decrease in accuracy for the end of both turns was most likely due to participants changing their walking angle after the turns. This was noted after the first 90° turn since some participants would walk on a diagonal across the second straight area, perhaps to turn in their preferred direction for the first 180° turn (e.g., if they start on the right after the turn but want to go around the 180° turn pylon on the left). This diagonal path made selecting the endpoint of the turn difficult since a distinct angle change did not occur (i.e., was the person still turning or walking along the second straight path?). This could also occur after the first 180° turn if the participant completed the 180° turn toward the inside of the circuit and then had to walk toward the outside of the first cone to complete the second 90° turn. At the end of the second 90° turn, participants may continue to turn slightly to prepare themselves for the 180° and sit-down tasks, therefore decreasing the distinction between the second 90° turn and 180° turn tasks.

The first 180° was classified with a higher Accuracy_AD_ (80% for the beginning of the subtask and 92% for the end of the subtask) than the second 180° turn (84% for both the beginning and end of the subtask). The lower Accuracy_AD_ for the end of the first 180° subtask was likely due to a similar occurrence as for the end of the first 90° subtask, with participants walking diagonally across the straight way to prepare for the subsequent turn. With the second 180° subtask, participants often combined movements for different tasks, resulting in further ambiguity of start and endpoints. This difficulty in assessing the 180° turn due to surrounding tasks was also seen in other studies, such as [14], where a TUG test with a longer walking time was chosen as the basis for the study’s designed algorithm (5 m instead of 10 m), since this provided a “gradual transition” between the straight walking and turning, leading to easier evaluation.

### Limitations

While this algorithm presents promising insights into the performance of the trained machine learning algorithm, there are a few limitations to consider. The sample size, although larger than most TUG models discussed, should be increased to allow for a more robust model. A larger and more diverse dataset would enhance the reliability and validity of the algorithm, allowing for a better representation of the population under study. The homogeneity of the sample could also be a limitation. While the dataset in this study is representative of a typical sample of LLA patients commonly seen at the Rehabilitation Institute of the Republic of Slovenia Soča, it comprises primarily similar samples, potentially limiting the algorithm’s ability to discern subtle variations or patterns present in more diverse populations. In future research, a new L test dataset with lower-limb amputees could be collected and used to test the model on unseen data. Addressing these limitations could advance the applicability and generalizability of this algorithm and similar algorithms in a clinical setting.

## 5. Conclusions

A random forest-based algorithm was developed to segment L test subtasks for application to lower-limb amputee populations. This classifier successfully identified transitions between subtasks within one step for most lower-limb amputee participants. Since all data collection was conducted on a smartphone, this approach could be integrated into a smartphone app that can provide analyses at the point of patient contact. Future research will expand the dataset and explore other deep learning models to improve classification results.

## Figures and Tables

**Table 1:** Able-bodied and amputee participant age and sex characteristics.

Age	Sex	Able-Bodied	Amputee
18–29	Male	3	0
	Female	9	1
30–39	Male	0	0
	Female	1	1
40–49	Male	0	0
	Female	0	4
50–59	Male	2	3
	Female	4	3
60–69	Male	1	8
	Female	1	1
70–79	Male	0	1
	Female	0	0
80–89	Male	0	3
	Female	0	0

**Table 2:** Amputee participant characteristics.

Characteristic	Number of Participants
Congenital	1 (4%)
Injury	5 (20%)
Peripheral Vascular Disease	5 (20%)
Diabetes Mellitus	9 (36%)
Other	4 (16%)
Unilateral	23 (88%)
Bilateral	2 (8%)
Ankle Exarticulation	2 ^1^ (8%)
Transtibial	22 ^1,2^ (81%)
Knee Exarticulation	2 (8%)
Transfemoral	1 (4%)
No Aids	20 (80%)
Single Crutch	5 (20%)

^1^ One participant had a bilateral amputation—ankle exarticulation and transtibial, counted in both categories; ^2^ one participant had a bilateral transtibial amputation, counted twice in the transtibial category.

**Table 3:** Ground truth earliest and latest instance times (by percent of total test time) for all participants (able-bodied and LLA).

Subtask	Earliest Instance (%)	Latest Instance (%)
Stand-Up	0	31
First 90° Turn	10	42
First 180° Turn	36	63
Second 90° Turn	61	84
Second 180° Turn	72	97
Sit-Down	76	100

**Table 4:** Accuracy_AD_ and corresponding tree, feature, and direction of analysis results for each subtask from the GridSearchCV function.

Subtask	Accuracy_AD_ (%)	Trees	Number of Features	Direction of Analysis
Start of Stand-Up	96	250	5	Forward
End of Stand-Up	96	250	50	Forward
Start of First 90° Turn	92	1000	10	Forward
End of First 90° Turn	76	1000	10	Forward
Start of First 180° Turn	80	500	25	Forward
End of First 180° Turn	92	500	75	Forward
Start of Second 90° Turn	92	500	5	Reversed
End of Second 90° Turn	72	500	5	Reversed
Start of Second 180° Turn	76	250	5	Reversed
End of Second 180° Turn	84	250	5	Reversed
Start of Sit-Down	92	1000	75	Reversed
End of Sit-Down	92	1000	75	Reversed

**Table 5:** Top 10 features for each subtask using the ANOVA F value.

Stand-Up	First 90-Degree Turn	First 180-Degree Turn	Second 90-Degree Turn	Second 180-Degree Turn	Sit-Down
Linear Acceleration X Minimum	Azimuth Range	Azimuth Range	Azimuth Range	Azimuth Range	Pitch Maximum
Pitch Maximum	Azimuth Standard Deviation	Azimuth Standard Deviation	Azimuth Standard Deviation	Azimuth Standard Deviation	Pitch Mean
Linear Acceleration X	Gyroscope Y Range	Azimuth Variation	Azimuth Variation	Azimuth Variation	Pitch
Pitch Standard Deviation	Gyroscope Y Standard Deviation	Gyroscope Y Maximum	Gyroscope Y Minimum	Gyroscope Y Range	Pitch Range
Pitch Range	Azimuth Variation	Gyroscope Y Range	Azimuth Kurtosis	Gyroscope Y Standard Deviation	Pitch Standard Deviation
Gyroscope X Range	Linear Acceleration Y Standard Deviation	Linear Acceleration Y Standard Deviation	Gyroscope Y	Gyroscope Y Variation	Gyroscope X Range
Gyroscope X Standard Deviation	Linear Acceleration Y Range	Linear Acceleration Y Range	Gyroscope Y Mean	Gyroscope X	Pitch Minimum
Linear Acceleration X Mean	System Time	Gyroscope Y	Gyroscope Y Maximum	Linear Acceleration X Minimum	Gyroscope X Standard Deviation
Linear Acceleration X Range	Gyroscope Y	Gyroscope Y Standard Deviation	Roll	Linear Acceleration X	Pitch Variation
Pitch Mean	Gyroscope Y Maximum	Gyroscope Y Mean	Roll Minimum	Linear Acceleration X Mean	Gyroscope X Variation

**Table 6:** Overall accuracy, sensitivity, and specificity results for all subtasks.

Subtask	Accuracy (%)	Sensitivity (%)	Specificity (%)
Stand-Up	95	92	100
First 90° Turn	97	77	100
First 180° Turn	98	87	98
Second 90° Turn	85	75	95
Second 180° Turn	88	85	99
Sit-Down	98	77	100

## Data Availability

The original contributions presented in the study are included in the article, further inquiries can be directed to the corresponding authors.
